# RETRACTION: Long Non‐Coding RNA HCG11 Modulates Glioma Progression Through Cooperating With miR‐496/CPEB3 Axis

**DOI:** 10.1111/cpr.70252

**Published:** 2026-06-22

## Abstract

**RETRACTION**: ChenY. , BaoC. , ZhangX. , LinX. , HuangH. , and WangZ. , “Long Non‐Coding RNA HCG11 Modulates Glioma Progression Through Cooperating With miR‐496/CPEB3 Axis,” Cell Proliferation 52, no. 5 (2019): e12615, 10.1111/cpr.12615.31310044 PMC6797506

The above article, published online on 16 July 2019 in Wiley Online Library (wileyonlinelibrary.com), has been retracted by agreement between the journal Editor‐in‐Chief, Qi Zhou; and John Wiley & Sons Ltd. The retraction has been agreed following concerns raised by a third party. An investigation identified duplication of the Cyclin D1 bands in the western blot presented for the U87 cells in Figure 6C with bands published earlier in another article by a different group of authors. The duplicated bands were also used to represent a different protein. The authors did not respond to requests to address the concerns and did not provide the original data. The editors therefore consider the results and conclusions reported in this article to be unreliable. The authors did not respond to our notice of retraction.

**RETRACTION**: Y. Chen , C. Bao , X. Zhang , X. Lin , H. Huang , and Z. Wang , “Long Non‐Coding RNA HCG11 Modulates Glioma Progression Through Cooperating With miR‐496/CPEB3 Axis,” Cell Proliferation 52, no. 5 (2019): e12615, 10.1111/cpr.12615.31310044 PMC6797506

The above article, published online on 16 July 2019 in Wiley Online Library (wileyonlinelibrary.com), has been retracted by agreement between the journal Editor‐in‐Chief, Qi Zhou; and John Wiley & Sons Ltd. The retraction has been agreed following concerns raised by a third party. An investigation identified duplication of the Cyclin D1 bands in the western blot presented for the U87 cells in Figure 6C with bands published earlier in another article by a different group of authors. The duplicated bands were also used to represent a different protein. The authors did not respond to requests to address the concerns and did not provide the original data. The editors therefore consider the results and conclusions reported in this article to be unreliable. The authors did not respond to our notice of retraction.

